# Creating a platform for costless personalization in clothing

**DOI:** 10.3389/frma.2022.990352

**Published:** 2022-09-06

**Authors:** Shane Greenstein

**Affiliations:** Harvard Business School, Boston, MA, United States

**Keywords:** digital printing, platforms, co-invention, innovation, commercialization, technology, print on demand clothing, chicken-egg problems

## Abstract

This study analyzes the role of co-invention in the creation of a platform for print-on-demand-clothing, or PODC. Co-invention is the invention of a new business process to complement new technology, and turn it into a valuable commercial service. PODC copies a design onto clothing with immaterial effect on the cost, and irrespective of the scale of the batch. In its modern form, PODC extends to more than two dozen different pieces of clothing and other items, enabling buyers to personalize clothing with any art. The digital printing machines used in PODC contain numerous technical inventions, while the electronic commerce platform contains the important business processes. The study examines a pioneering PODC platform from Threadless, and analyzes how this new platform emerged from a sequence of co-inventions. The study highlights the level of discretion given to graphic artists to foster trust with the platform, and it shows how a hierarchy of business process co-inventions overcame the coordination issues inherent in building a large scale and new multi-sided platform.

## Introduction

Inventions of new business process turn new technology into valuable commercial services. Invention in business processes can redefine job tasks, such as daily assignments and skill requirements, and alter lines of authority, such as discretion over decisions and procedures for resolution of conflicts. Inventions of business processes are called co-inventions to distinguish them from the initial invention. The study analyzes the co-invention for creating a platform for print-on-demand-clothing, or PODC.

Today PODC illustrates the marriage of technical invention and business process inventions. PODC copies a design onto clothing with immaterial effect on the cost, irrespective of the scale of the batch. In its modern form, PODC extends to more than a dozen different pieces of clothing—from shirts and sweatpants to shoes, socks, and masks—yielding an explosion in unique combinations of sizes, items, and designs. It also extends to many items—such as coffee cups, shower curtains, rugs, buttons, and blankets. PODC enables a buyer to personalize any item and size with any art of their choosing. The buyer can get matching shoes, shirts, hoodies, leggings, socks, and masks printed with the same design, or get the same sweatshirt in a range of designs. These combinations also come at no added cost, albeit modern electronic commerce adds cost for shipping to the buyer.

The digital printing machines used in PODC contain numerous technical inventions, while the electronic commerce platform contains the important inventions in business processes. The platform matches clothing manufacturers, graphic artists, and buyers. Whereas, digital printing machines has received attention among industry news publications, the reorganization of platforms has not received attention. The goal in this study is to analyze this neglected topic, and examine the role of business process inventions in making PODC viable at scale. The research questions focus on the earliest efforts, and are seemingly simple: What prompted pioneering in new business processes to support PODC? What co-inventions enabled the pioneers in PODC to achieve a high volume of transactions and low cost PODC?

We study co-invention, specifically, at Threadless, a company that pioneered high-quality PODC within a platform for graphic artists and buyers. Threadless' PODC platform takes the form of a service called Artists Shops. This is not Threadless' first effort at pioneering new services for graphic artists and buyers, as the firm also pioneered a commercially successful platform that crowdsourced graphic art for t-shirts. Artists Shops differs in both scale and breadth. It has more than a hundred thousand partners among graphic artists, and a similar magnitude of partners among holders of trademarks. Artist Shops arranges to make both clothing and items. The combinations of art, items, and sizes realizes the promise of PODC, yielding an explosion of unique items for sale with no change in costs and prices, nor any loss in operational flexibility or delivery speed.

Some aspects of Artist Shops reflect the familiar elements of a three-sided business platform involving graphic artists, manufacturers, and buyers. That the platform and manufacturer share the activities affiliated with order-fulfillment processes is another familiar feature. It also operates in a setting, apparel, which is competitive. All these features make Threadless a good example for study because the unit costs, prices, and margins per product did not change as Artist Shops grew. As will become apparent, Artist Shops could not succeed unless it achieved high volumes, which happened as purchases increased, and that depended on the matching of designer, buyer, and manufacturer. That frames the focal question for analysis: what co-invention did Threadless create to put together a platform that matches so well at a large scale?

These co-inventions are interesting in their own right because they were not obvious, at least at the time they were invented. Innovating at the junction between the responsibilities of the graphic artists and the platform, Artist Shops offers control over most of the elements of the transaction to graphic artists, including intellectual property. It also affords the artist the option to cede discretion back to the platform to act on their behalf (in a service called Managed Shops). In practice, graphic artists often cede discretion over a wide range of decisions to Threadless, which enables the company to choose a manufacturer, govern features of the transaction with buyers, and determine features of distribution, including the price at which the item sells and the timing of sales. Because this model is popular with graphic artists, Threadless delivers a large volume of orders to dozens of manufacturers in digital printing, and across a wide array of items. That scale enables zero costs for adding each new design, which Threadless then passes on to buyers.

New electronic commerce platform must achieve large volumes of transactions to offset the idiosyncratic fixed costs of operating data bases, order fulfillment, quality control, and specific back-end processes. Yet, it would be reductionist to view the development of Artist Shops as just a story of low margins at high volumes covering fixed costs. The narrative focuses on a phenomenon for which platform analysts rarely get an inside look—namely, the “chicken-egg problem” at an early moment in the platform's creation. It is difficult to be both a small and new platform, especially when each party has distinct interests and disparate motives for participation. This study informs understanding of the general situation with analysis of a specific example: how did this new platform create, establish, coordinate, and sustain relationships with participants who benefit from participation by the others? How did this platform grow when the success of matching depended on a large scale of participation? By studying one example of how one firm resolved this dilemma, the analysis highlights the sequence of co-inventions that accumulated to yield a new platform.

The narrative suggests a hierarchy of business process co-inventions determines the order—starting with those complementary to the usage of the invention in digital printing, moving next to those related to the needs of a key partner, graphic artists, and ending with the establishment of a new platform for governing the relationship of buyers and sellers. Further iterations scaled the platform in terms of more breadth of items, and, simultaneously, more participation of graphic artists. That hierarchy corresponds with co-inventions that started by orienting toward cost savings from adoption of a new invention. It delayed riskier co-inventions that support new product development with an unknown scale of demand.

### Contributions to literature

No analysis of the creation of new platforms has organized its analysis around co-invention, a concept that originates from the literature on *adoption* of enterprise-wide computing platforms. In the earliest studies of co-invention, experimentation and discovery by users fostered co-inventions. The concept has been used in analysis of a wide variety of settings. For example, it contributes to analyzing the speed of the transition between usage of mainframe computers and client-server systems (Bresnahan and Greenstein, 1996), the transition to usage of internet-enabled administrative processes (Forman, 2005; Forman et al., 2005), the change in industry leadership during digitization of administrative tasks (McElheran, 2015), the cost savings to hospitals during the transition to electronic medical records (Dranove et al., 2014), and the rise in productivity at manufacturing establishments during the early transition to cloud computing (Jin and McElheran, 2015). These prior studies analyze variance across adopters in their co-invention activity. In contrast, this study analyzes *variance over time* in one supplier's co-invention actions. That might seem minor at the surface, but it draws attention to a large gap in the literature. While nothing precludes co-invention at suppliers (Bresnahan and Greenstein, 2001), the literature has largely not explored the possibility. In this instance, one firm both adopts and supplies while co-inventing. Co-invention accompanied the adoption and usage of digital printing, and then another set of co-invention created a platform for matching digital printers, graphic artists, and buyers.

Many prior studies of platforms have stressed the challenges of overcoming frictions created by anonymity and distance inherent in electronic commerce (Goldfarb and Tucker, 2019). Trust between buyer and seller emerges from clever market design, such as reputation systems, and from other tools that facilitate repetition of transactions between buyer and seller (Levin, 2013; Luca, 2017). A novelty in this study are the platform's rules in fostering trust between the platform and its partners, graphic artists. Threadless created a platform that enables multi-homing by its graphic artists. It gives them options and discretion, which earns a high level of trust. That co-inventive change in rules contributed to generating large scale participation from graphic artists. Interestingly, such discretion is not normally regarded as consistent with a platform's interests. The prevailing view is that the freedom to multi-home hurts platforms (Zhu and Lansiti, 2019).

The narrative follows Threadless as it evolves from a firm managing a crowd-sourcing platform into a firm managing two platforms, the latter oriented around PODC. The contrast between the old and new platforms raises themes reminiscent of those in the literature on “disruption” (Christensen, 1997; Gans, 2016). In the classic narrative, the evolution of a technology from low quality to high quality leads to devaluation of business processes in an established business. Elements of that narrative appear in this narrative, and this begins as an unsurprising element of the analysis. For example, digital printing began as an input into a low quality product and improved over time and became an input for a high quality product. Though the rate of improvement was challenging to forecast, the established businesses, Threadless, recognized the direction of change, and surmised that it contained the potential to devalue established business practices. That motivated management to initiate experiments to gain insight into digital printing. This narrative takes a surprising turn, however, and does not yield a standard story for the disruption literature. These experiments did not encounter resistance that doomed the efforts, and the established firm did not begin on a path of decline. Instead, Threadless succeeds in creating novel value with new co-inventive activity, and that supports business renewal with a new platform.

More narrowly, this study also contributes to understanding innovation in the supply chain for apparel. It contrasts with strategies that focus on *simplifying* product assortment or reducing complexity of supply chains (McKinsey and Company, 2021). PODC widens the breadth of product assortment and manages the complexity within a platform, and does not sacrifice flexibility and costs.

When compared against the two billion t-shirts sold in the U.S. each year or the 100 billion dollars of global sweatshirt sales, PODC cannot increase the aggregate economy. Yet, this study rejects the view that interprets PODC as an innovation that solely enables “business stealing.” In business stealing, the increase in sales at a firm such as Threadless decreases the sales at another (nameless) firm. This study interprets PODC through a wider lens. It views PODC as part of a broader trend in the increasing prevalence of *digital dark matter* (Greenstein and Nagle, 2014). Digital dark matter arises when inputs cost zero. This topic has received attention in research covering open innovation (Altman et al., 2014) and open source software (Keller et al., 2018; Robbins et al., 2018; Marciano-Goroff et al., 2021). Consistent with the literature in digital dark matter, conventional economic tools mischaracterize PODC because neither improvement in input nor improvement in user satisfaction is measured by any government-sanctioned statistic, nor by any conventional cost-accounting procedure inside a firm. Yet, the new platform organizers, manufacturers, and graphic artists are better off, at least as revealed by their continuing participation in the platform. The buyers are better off too, at least by their revealed preference, in that they bought the artful piece of clothing and are therefore happier with the product than they would be with another one. Just as Coco Chanel once famously said, “The best color in the whole world is the one that looks good on you,” the purchased PDOC product is the best one in the world for each buyer. All of this implies that the standard GDP measurement and accounting methods are inadequate for measuring the gains to suppliers and buyers from costless personalization with art.

### Outline

The essay presents events in chronological order in Section The history of threadless' transition to artist shops, which describe Threadless' crowd-sourcing platform and management's response to the distant threat posed by digital printing. In the next section, we analyze Threadless' response, and its expectations for cannibalization and economies of scope. Next, in Section Artist shops from a variety of perspectives, we describe PODC as found in Artist Shops. Section Creating PODC in artist shops takes a step back, and identifies and analyzes the co-invention required to modify the old platform. It also analyzes several opt-in features of Artist Shops that illustrate interesting and important facets of co-invention related to the platform ceding control to graphic artists.

## The history of threadless' transition to artist shops

### Pioneering a crowdsourcing platform^1^

**Figure 1:**
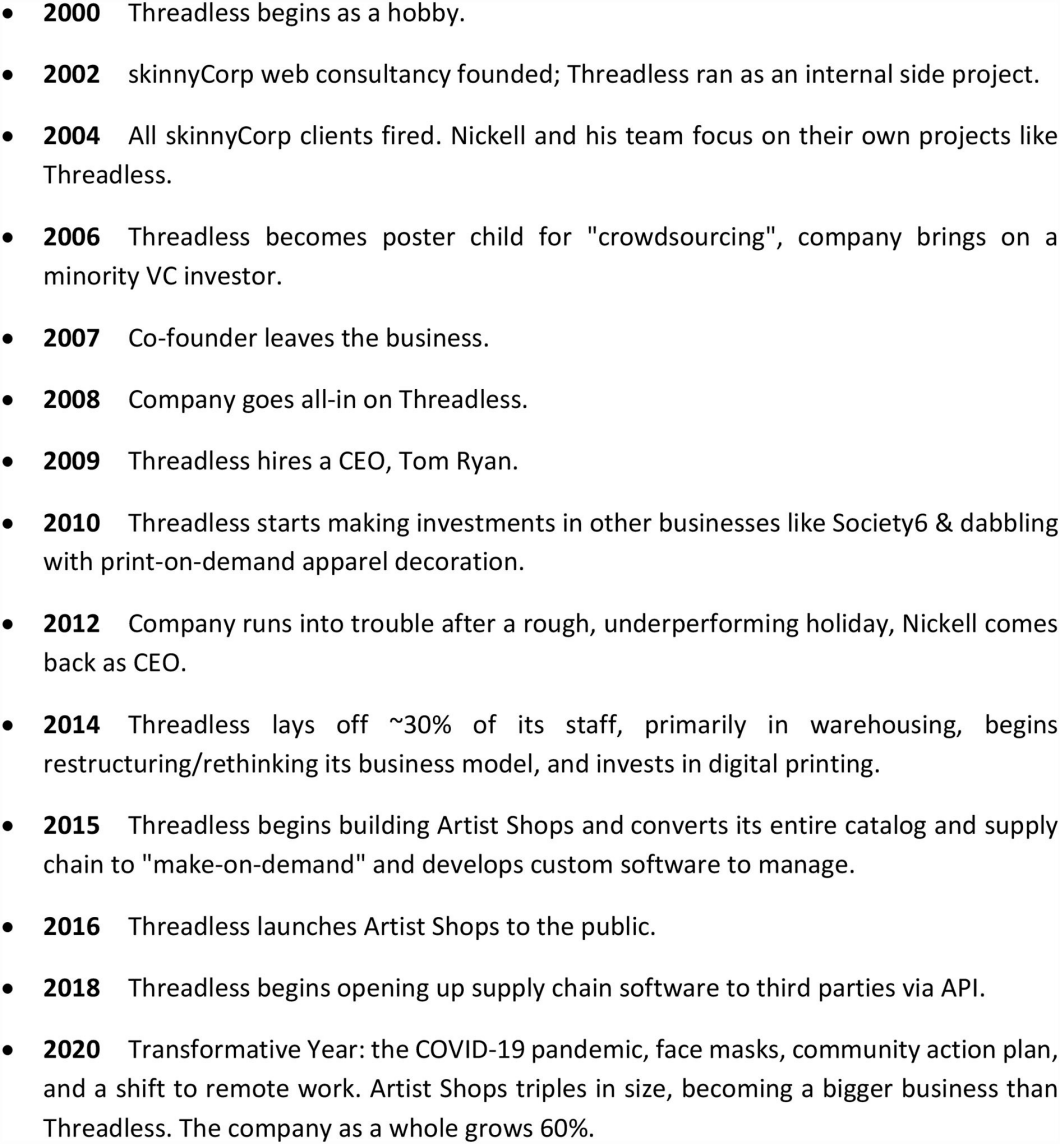
Threadless Historical Timeline (Greenstein et al., 2021).

Founded in late 2000 by Jake Nickell (Chief Executive Officer), Jacob DeHart (Chief Technology Officer), and Jeffery Kalmikoff (Chief Creative Officer), Threadless started as a side project. The company grew into a pioneer of crowdsourcing, supporting a platform that helped a diverse community of graphic artists produce unique designs for millions of online customers. After several years, Threadless became successful enough to move into a 25,000 square foot warehouse and bring in tens of millions of dollars of revenue per year. Threadless became successful enough to move into a 25,000 square foot warehouse and bring in tens of millions of dollars of revenue per year (see Figure 1).

**Figure 2:**
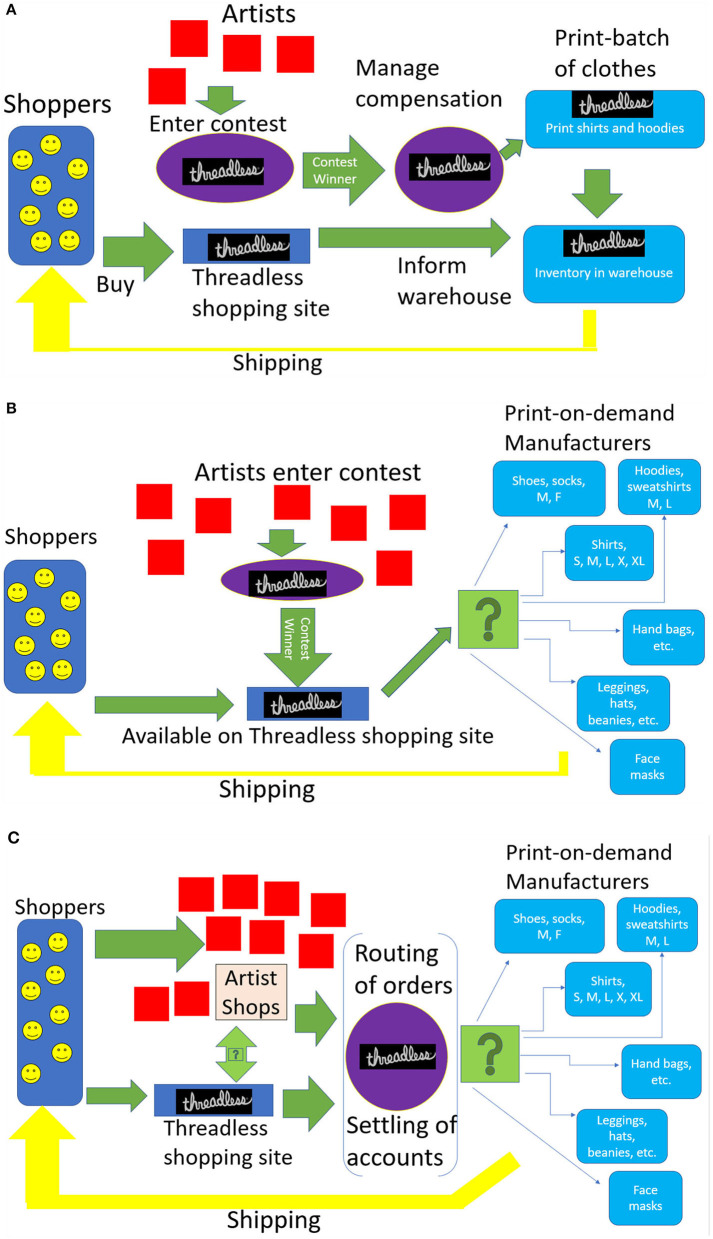
**(A)** Original threadless crowdsourcing operating model. **(B)** New threadless crowdsourcing operating model. **(C)** Artist shops platform operating model.

The platform was based on weekly design competitions, where graphic artists submitted their designs for t-shirts and sweatshirts. Other artists and potential customers voted on submitted designs. Threadless printed the most popular of these submissions and sold them in an online store. In the earliest model of this business, once the winning designs sold out, they were replaced by newer designs. In later versions, some of the winning designs underwent additional print-runs after they sold out. The operations behind Threadless' crowd-sourcing platform is visually represented in Figure 2A.

Threadless' management nurtured a sense of community among the artists and buyers. For graphic artists, the design challenges and voting process served as an inspiration. Even when an artist failed to win, they could gain insight from the feedback received. Sometimes the contests were open ended, and sometimes they were organized around themes. Such challenges also served to make goals concrete and channel creative thinking. Many artists enjoyed sharing their work with a community of fellow artists and art lovers.

In the language of modern platform economics, Threadless invested in motivations for one side of its business, namely, graphic artists, but the motivations were not entirely monetary. Rather, they mixed extrinsic, intrinsic, and pro-social motivations. Threadless made efforts to nurture all three motives and support them. In the language of the platform literature, Threadless' relationship with the artistic community was among its most valuable intangible assets^2^.

From the beginning, Threadless' decision to commit to high quality printing shaped the tension between scale, cost, and color. Later experiments with PODC generated both scope economies and some cost reduction^3^, but early on, Threadless used only superior materials and processes. Choosing screen printing for the production process incurred higher costs not only from the ink, equipment, and length of set-up time required Farag (2021), but also from the significant amount of time (i.e., more than a day) required to create a new screen for each new design. To lower costs, Threadless placed strict limits on graphic artists, such as requiring no more than four colors, which reduced the error rate and reduced the time and expense of setting up a machine to print the shirts. Obviously, per unit costs became lower as the order quantity increased.

Threadless' crowdsourcing platform also generated some tension between scale, cost, and color, with regards to how many shirts the management should make for each design in a print-run that won a contest. Over the long run, the total revenue had to exceed variable costs enough to yield a gross profit per unit, and the gross profit had to pay employees and other expenses, such as warehousing. The price tended to be outside the firm's control, because it competed against so many other artistic products and other pieces of clothing. With normal pricing and sales, prices approximately landed at twice the cost of materials^4^. The foregoing turned the question about the size of print-runs into a cash-flow issue. With the run rate of a typical machine, the number of new prints per week was initially set at 2–3 per week.

Finally, Threadless also had to deal with up-front costs. Before any revenues for prints were collected, Threadless first incurred costs for two expenses: paying the artist for the winning design^5^ and purchasing materials to create shirts. In the early days, Threadless' management weighed a number of considerations when choosing the volumes for its print runs, but principal among them was the question of how long it would take to recover enough revenue to pay for the cost of a print run.

Threadless always encountered a difficult forecasting problem for each design and paid directly for forecasting errors in both directions. When the company printed copies of a design that *did not* appeal to many buyers, Threadless would hold unsold inventory and never generated enough revenue to cover expenses. When the company sold large quantities of a new design that *did* appeal to buys, it could face a stockout. A stockout could prevent Threadless from selling otherwise profitable shirts, which framed questions about whether to incur the costs of setting up and organizing additional runs of a design. The latter problem was better than the former, because it was easier to address a stockout for a popular design with a new print run than to sell an unpopular design.

In light of those costs and risks, what was the best number of shirts to print on a run for a new design? Should it be five hundred, two thousand, or ten thousand? One thoughtful approach would try to estimate expected sales, but how can demand for another unique design be estimated from the experiences of other unique designs? Almost by definition, Threadless started its forecasts for each unique design with little information; however, crowdsourcing played an essential role in helping the firm anticipate demand. Although it wasn't perfect, crowdsourcing turned out to be far better than merely guessing when a unique design might appeal to an online buyer's sense of humor or unusual sense of aesthetic. Sometimes crowdsourcing's high vote totals and enthusiastic comments provided additional indications that that the appeal would be strong^6^. Altogether, this process helped Threadless keep unsold inventory lower, which translated into faster revenue and lower inventory costs in the long run.

Threadless' crowdsourcing model evolved into a practiced machine, and the company tended to settle on printing approximately five hundred shirts per size, gender, and design on the first run^7^. With each design printed on at least three sizes for two genders, the result was potentially thousands of shirts in inventory for each design^8^, so Threadless invested in a warehouse operation and employed experienced workers. At its largest in 2014, the warehouse held approximately nine thousand t-shirts and sweatshirts for six different sizes and two genders. At its peak, Threadless selected approximately three winners per week and 150 designs per year.

### Initial threat from print-on-demand

Why would a firm with a unique and successful crowdsourcing business consider PODC? In the early 2010s, compared to Threadless' high-quality products, PODC yielded low-quality print designs that were not free of errors. Many PODC items were limited to black and white designs or low-quality color ink that faded after washings. Available through companies like Café Press, many of Threadless' buyers did not consider PODC to be substitutes, nor did most of the graphic artists who participated on Threadless' contests.

Contrary to a classic case of “disruption” from improvement in a low-quality rival (Christensen, 1997; Gans, 2016), PODC displayed no clear trend for how fast the quality would improve. There was no forecast data by which a future response was required, nor any urgency from an imminent date at which high-quality would become a threat. Nevertheless, the presence of a low-quality alternative evoked a set of future questions, and Threadless' management talked openly about them Greenstein et al. (2021).

Thinking about future scenarios does not necessarily generate action. In this case, a change in management shaped the timing of Threadless' first actions. Like many startups, Threadless had experienced turnover among its founders, though one of them, Jake Nickell, continued to hold equity ownership control over it. In 2007, Jake Nickell had backed away as CEO and a new one was hired. In fact, all the founders had left day-to-day decision making after a few years. But after a poor holiday season in 2011, Nickell returned in 2012 to daily management as CEO.

It's an overstatement to say Nickell came back to turn around the business, as Threadless was not in a free-fall, and it is an overstatement to say new management brought a new outlook, since he had helped establish many of the key elements of the business. Rather, his return led to a full reconsideration of the business processes and services, and those reconsiderations coincided with the presence of low-quality PODC. The timing, though inadvertent, triggered new experiments.

What PODC scenario concerned Jake Nickell? He wondered what would occur as quality improved. He believed Threadless risked losing artist participation in design contests, as well as buyer interest. He considered the graphic artists first: An artist's chance of winning was low in a Threadless contest, though it was mildly higher for experienced artists. That meant low- to medium-quality printing might appeal to graphic artists who had lost many of the Threadless contests. Or, in the language of platform economics, it might generate more “multi-homing” by graphic artists.

Next, he considered the buyers: Other companies could produce simple designs at almost the same costs as Threadless. At some point, buyers would not notice the difference in designs or quality, so the potential sales could come at the expense of Threadless. Or, in the language of platform economics, it might reduce the elasticity of demand for Threadless' products.

Taken together, if either of these disturbing concerns about graphic artists or buyers were realized, it would hurt Threadless' crowdsourcing platform. In that sense low-quality PODC defined a distant but realistic threat.

A related short-term motivation also prompted action. Digital printing potentially eliminated batch production or reduced it greatly from the limitations imposed by screen printing. The absence of batch production could change many aspects of the crowdsourcing model, such as the level of inventory holdings and concomitant working capital. That views digital printing not as a threat, but as a cost-saving invention, and an opportunity to alter internal processes for printing.

Nickell began to authorize experiments, aimed principally at whether and how to transition away from screen to digital printing. These efforts began as a defensive response to eventual but distant threats, and came with the potential for cost savings in the existing crowd-sourcing model. Nickell did not begin within a forward-looking strategy to expand the business with new services. The expansion developed later.

Rephrasing, at the outset, we see the beginning of the hierarchy of co-invention. Nickell started with an experiment with visible and short term benefits—i.e., digital printing could reduce costs for the existing business. Later co-invention was deferred until this set could be understood.

To summarize, the initial actions were exploratory, defensive, narrow, forward looking over a short time horizon, and oriented around understanding how digital printing worked in their crowdsourcing platform. They would undergo a marked change only after management learned lessons about how to operate digital printing at scale and across a broad array of clothing items.

### Transition to digital

Altogether, the invention of PODC in an electronic commercial platform took 4 years. Starting from Nickell's return as CEO in 2012, the first successful implementations of digital printing were up and operating regularly in 2014. By 2016, Threadless completed its transition to digital printing for *all* of its printing. In the meantime, Threadless had been experimenting with its business processes and, also in 2016, introduced the Artist Shops. Unlike its earlier efforts with the technical innovation of digital printing, Threadless' Artist Shops were not the result of defensive efforts, nor were they narrow in scope.

Why did Threadless undertake the efforts with Artist Shops when its crowdsourcing business processes were successful? Addressing that question requires analysis of intellectual property and the order-fulfillment process. In contrast to the establishment of Artist Shops, Threadless altered each of these business practices as part of its defensive efforts in the face of PODC. Both the changes in intellectual property rights and order-fulfillment processes ultimately influenced Threadless' initial experiments with PODC and can be interpreted as key co-invention activities.

#### Intellectual property

In its original model, Threadless required the graphic artists to sell the rights to their design to Threadless for a fixed fee. The fee was the reward for winning a contest. After returning as CEO, one of Nickell's first acts transitioned the firm to a new compensation structure in which the artist retained intellectual property and Threadless paid them a royalty for every piece of clothing that used their image. In comparison to the old structure, this new structure rewarded the artists whose product sold in large quantities; however, it also removed the minimal rewards that all artists could expect, which raised the risks for inexperienced artists. The new structure also lowered Threadless' risks for holding inventory because it lowered the upfront cash payments. It came with the risk that a successful artist could take the intellectual property for their art to another printer.

Interpreted through the lens of a standard model of platforms, this change could be seen as a change to induce more participation from one set of graphic artists, namely, the most talented graphic artists. It came with the risk that it made it easier for a successful graphic artist to multi-home. The new structure was regarded as closer to “fair” by successful artists, and, on net, it did result in more participation, as intended.

That interpretation is incomplete, however. By 2012 Threadless owned all the designs it had accumulated from years of contests. Nickell sensed resentment over these holdings. Despite the concerns of his legal counsel and other executives, Nickell chose to change all of Threadless' holdings. The firm contacted all the original artists and sold their design back to them.

The drawback of transferring ownership back to artists was the monetary cost, as well as the administrative hassle. In effect, Nickell's decision obligated Threadless to pay compensation in the future to past contributors when legally nothing was owed. It also gave graphic artists complete control over their own back-catalog, which enabled them to multi-home to a greater extent. This cost came with a symbolic gain, however, and these gains were less extrinsic and more prospective. First, some of the same artists who had won contests in the past still participated in 2012, and a few of them were responsible for some of the best-selling designs. Although returning the ownership back to the artists did not guarantee their continued participation, it eliminated a source of resentment. Second, it was readily apparent to many of the graphic artists that Nickell faced no obligation to take this action. This vested the action with symbolism as a gesture of goodwill.

It might be tempting to interpret such behavior as emerging from a calculated attempt to enforce an implicit and legally unenforceable contract. It is, however, simpler to interpret it as a gesture of trust borne from a principled stand. Supporting graphic artists was a central mission of the company. Nickell' gesture was indicative of his general honesty and a plainspoken, forthright approach to business, as well as his empathy with the graphic artistic community's perception and outlook. That earned the trust of many graphic artists.

#### Restructuring order fulfillment

Threadless next changed its sales and order-fulfillment to accommodate digital printing in its crowdsourcing business. They began to experiment with holding less inventory. This set them on a multi-year process of gradually reducing the size and staffing of the warehouse. In 2014, for example, Threadless laid off 30% of its staff, primarily in warehousing. A few years later they would get rid of all their warehousing space and staff.

Order fulfillment is a central feature of electronic commerce, and developing a reputation with buyers for timely delivery a key aspect of the business (Levin, 2013). While digital printing reduces costs, it comes with the increase in dependence on the performance of manufacturing partners. This increases risks for Threadless if partners do not perform. Interestingly, it comes at a time when the largest provider of electronic commerce, Amazon, has invested heavily in facilities for large scale order fulfillment.

On their web page Threadless also undertook another long-term effort to restructure their display and sales processes. Initially Threadless had designed its site to sell to a dedicated buyer community that came to browse the new artistic winners each week. The scale of the available number of new designs no longer made that a viable approach. Digital printing enabled Threadless to increase the colors of shirts it could sell (i.e., to thirty), and the types of shirts on which it printed (i.e., regular and v-neck, sweatshirts and hoodies, etc.). Accordingly, the company began a transition to a more search-based consumer, one who looked through a large quantity of stock keeping units (SKUs).

Modifications were undertaken by an internal staff of programmers, and their skills and duties extended across the entire supply chain, specifically, web design, order tracking, payments, and shipping. They maintained technical road maps for short- and long-term plans in these processes, and they remained constantly occupied with additional projects for improvements^9^.

Overall, the efforts to incorporate digital printing into Threadless' crowdsourcing business, as represented by the transition between Figures 2A,B, brought the management closer to understanding the technical possibilities of PODC and, as will be shown subsequently, provided the inspiration to construct a new innovative service, the Artist Shops.

### Complements as co-invention

The changes in intellectual property and order fulfillment between the old crowdsourcing model and the new (see Figures 2A,B) could not have occurred without co-invention. These changes were complements to digital printing, and they made PODC viable and valuable. In other words, the opportunity posed by PODC motivated the changes in business processes in that rather than owning the art, Threadless licensed it from the artist. There was no secondary market for such innovations, no easy ways to value those co-invented intangible assets (e.g., sense of community, loyalty), and no external source of validation telling Threadless' management that they had undertaken the best path. Instead, all such actions came with a great deal of uncertainty surrounding PODC's long-run viability and profitability. In this sense it is less surprising Threadless' first actions were the least risky, and oriented toward understanding digital printing and reducing costs in its existing business.

That still leaves unaddressed why Threadless decided to invent Artist Shops, whose business structure is represented in Figure 2C. In 2020, Nickell gave an interview that explained the inspiration for Artist Shops. Threadless had pursued blending digital printing with their existing crowdsourcing platform, but such activity raised questions about developing new lines of businesses. They had experimented with PODC, such as partnering with or purchasing other firms who produced PODC in other types of clothing, but these did not amount to much^10^. As Threadless neared the completion of adapting digital printing to their crowdsourcing business in 2015, Nickell explained that they needed to do something big, and the “aha moment” came with the thought, “Wouldn't it be cool if artists could have their own branded store rather than being in a marketplace?”

This idea was along the lines of a Shopify version of digital printing, which didn't exist when Threadless first started out, but was available and understood by any producer in online retailing by 2015. At the time, Shopify was a service that allowed its licensees to launch their own online stores for almost any product. Such a service did not exist in a format that made it suited for graphic artists, but Nickell realized that filling that gap, if done well, would be consistent with Threadless' mission.

Consider a catalog of the operations necessary to build “a Shopify for graphic artists” and whether that displayed any overlap with the existing operations illustrated by Figure 2B. At first glance, many complementary activities were comparatively familiar. For example, Threadless already had built and maintained the databases and related software for supporting big data applications, and they had years of experience putting those applications to use inside an order fulfillment process. In addition, Threadless had just developed a new business process for distributing an exploding number of SKUs. Their existing business was, however, less complex and less geographically distributed than the one they were proposing to build. In short, there were overlaps of software operations and development, but the new processes potentially involved additional software as well, though much of additions were incremental and would not take long to develop.

The inherited customer base also shaped Threadless' outlook, both positively and negatively. The most positive aspect was the most fleeting. On the one hand, the existing customer base provided a potential solution to the Artist Shops' need for customers and it could serve as an attractive target for sales of the new services in digital printing. Moreover, its graphic artists, especially the most successful among them, could serve as the source for new art for Artist Shops. On the other hand, Nickell and his team worried that the presence of the customer base could give rise to cannibalization of sales. That is, they worried that the Artist Shops would merely generate sales at the expense of sales on the crowdsourcing platform, which, on net, would not yield additional revenues on par with the expenses and efforts required to develop the new service. More worrisome, the cannibalization could arise after Threadless used its most precious intangible asset, namely, its relationship with the graphic arts community. This was a potentially irreversible risk.

Those concerns did not deter them for two reasons. One, not convinced that their Artist Shop adaptation to crowdsourcing would be sufficient to respond to the distant threat posed by PODC when high-quality products were prevalent, they worried that more effort and innovation might be required. Two, their mission involved supporting graphic artists. Fulfilling that mission required them to go all the way in providing a new service, even in the face of cannibalization.

In summary, while defensive motives impelled Threadless to take the first cautious set of steps to learn about digital printing and adapt it to their platform, a later epiphany motivated the second set of riskier co-invention activities. Moreover, their motivation arose from a collection of mission-oriented strategic concerns, a combination of experiences while they built the processes to support digital printing, and observations about a business model (Shopify) that they adapted to fit their own products. Was this just lucky or luck favoring the prepared mind? While we have stressed the costs savings from overlapping business processes during this transition, and the realization of less cannibalization than anticipated, perhaps the key piece of good fortune was the continuing value of Threadless' key intangible asset, its reputation with graphic artists. Rather than facing a choice between its old and new businesses, Threadless, could operate for a time, turn prospective costs savings into visible business processes, and learn how to adjust its business processes to support creation of a new platform. As it happened, they were surprised by the positive response, which put their cannibalization concerns to rest.

## Artist shops from a variety of perspectives

Artist Shops are a multifaceted service, and that makes it challenging to identify all the co-inventions it contains. It is useful to describe Artist Shops from one of three perspectives—from the perspective of the graphic artist, the buyer, and the management at Threadless. Each perspective provides different insight into the co-inventions that make it successful.

### The graphic artist

**Figure 3:**
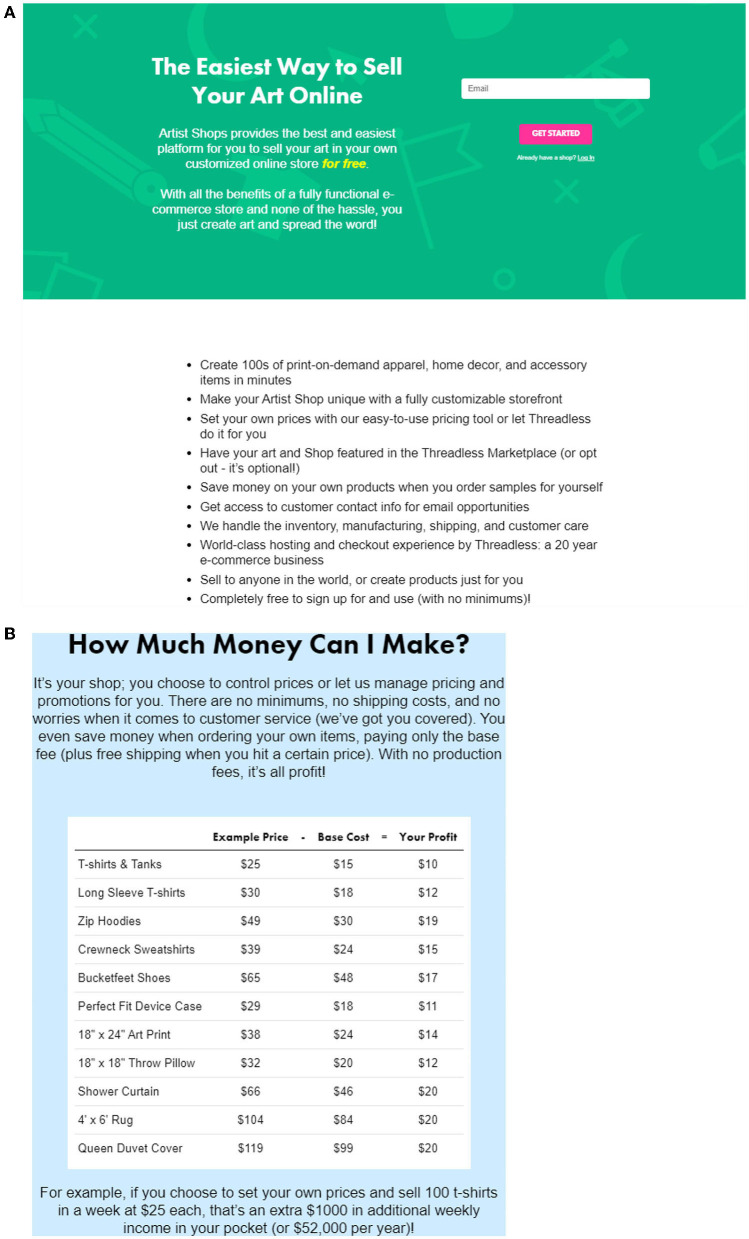
**(A)** Signup page for graphic artists. Artist Shops Sign-up Page, https://www.threadless.com/artist-shops/signup/art, accessed September, 2021. **(B)** Information page for graphic artists. Artist Shops Sign-up Page, https://www.threadless.com/artist-shops/signup/art, accessed September, 2021.

Artist Shops in 2020 allow all graphic artists to sign up for a store, create their own brand name, upload their art, and choose the specific products on which their designs are available to buyers. They then can start selling immediately and at no cost. Artists can expect compensation as the difference between the price and a base cost, which differs depending on whether the item is sold in the Artist Shop or by the Threadless website^11^. Typical compensation tends to be around 25% of revenue but varies with the price and baseline cost. As examples, Figures 3A,B provides screen shots of Threadless' explanation to graphic artists.

A graphic artist with a modest portfolio can expect to engage with the product proliferation enabled by PODC. For example, a seller who has thirty illustration designs might choose a range of apparel, say nine different apparel items in two genders, as well as a range of sizes, including children's sizes. For purposes of illustration, make it four sizes per item. That implies their shop would offer 30 × 9 × 2 × 4 = 2,160 SKUs for sale without having to carry any inventory. Each of those items does not compromise on style, fit, gender or other attributes of the clothing. In addition, if Threadless asked for it, and the artist provides permission, their designs can appear on the Threadless website^12^.

This service appealed to many graphic artists. For the basic service, Threadless did more than just provide a framework for displaying designs and the clothes. Threadless handled the payments after a sale, compensated all parties, ordered manufacturing, routed the shipping, ensured quality control, and mediated any disputes. Most artists perceived this as a valuable time saver for tasks they would otherwise not be able to perform to the same quality, or necessarily perform at all.

Threadless offered artists additional opt-in services. Among them was a service called Managed Shops, which an artist subscribed to for a small additional fee. With Managed Shops, Threadless set the pricing within the shop and ran promotional events, such as ads for a Memorial Day sale. By 2020, more than 95% of licenses for Artist Shops made use of this option (more below about this astonishing rate of participation).

Threadless also gave the most successful of the artists the option to let Threadless act as their agent and help artists go beyond online retail by getting their designs sold in brick-and-mortar stores. Threadless also could function as agent for a product using the artist's design, where the retail shop carried out the manufacturing and sales process^13^. Extending a similar logic into additional distribution channels, Threadless also could help its artists get their products into other online stores through a service they called *Virtual Catalogs*, which gave artists plug-ins for other online distribution outlets.

Notably, none of these services came with a requirement that the artist distribute their product exclusively using Threadless' services. This lack of exclusivity with each additional service infused artists with a sense of control and with the right to test the value of the service by observing experience. These offers presumed that artists managed their own brands and that the risks for the opt-in intermediary services fell on Threadless and not on the artist. Nickell commented, “If they don't like it, they can opt-out and do it themselves. But the proof is in the pudding—you earn more when you opt-in.”

One other type of artist engaged with Artist Shops, and they were initially unexpected by Threadless' management because they differed from the typical graphic artist with a portfolio of art to sell. This participant consisted of an organization with a few logos and related trademarked symbols. For this type of licensee—typically a corporate organization, a non-for-profit organization, or a school club—Artist Shop offered a convenient method for distributing shirts, jackets, blankets, and other items with the logo. The organization set up the shop and then distributed the website address to its members, who then purchased the items and independently received delivery at their doorstep. As of 2020, around one hundred thousand organizations had made use of the service for such purposes.

### The buyer

When buyers shop on an Artist Shop, they do not see the Threadless name unless they look carefully for it in the corner of a webpage. Instead, they see an online store that orients around the brand an artist wants to display. The online store provides a menu of optional designs and optional pieces of clothing on which to apply the design. After purchasing an item, the buyer receives a package at their residence some days after the order. This shopping experience reflects the standards for online commerce in 2020, with low frictions for search, display, ordering, payments, and delivery.

When buyers shop on the Threadless website, they may find some of the same items as found in the Artists Shops. The vast majority of the time these items will be listed at the same price as those listed in the Artist Shops (as when the artist has opted to let Threadless managed their shops). The buyer who finds these designs may compare them with other designs on the Threadless website with similar themes, moods, or elements. After purchasing an item, a buyer experiences the same order fulfillment process.

### The platform

Threadless first announced Artist Shops in 2015 and invited known artists to reserve their URLS in advance, before a full launch in 2016. The platform grew quickly and became the largest source of revenue for Threadless by mid-2020. Between 2015 and 2020, Threadless gained additional insight into this form of electronic commerce. In Figure 2C, we illustrate Threadless' operations.

Threadless investigated its cannibalization concerns soon after launching the Artists Shops and was relieved to confirm that most of the artists licensing Artist Shops were not participants in the crowdsourcing contests on Threadless' original site. Though they were surprised, they quickly discovered that these artists opted out of crowd-sourcing contests because they (1) wanted control of their own brand, (2) wanted to avoid direct competition with other artists, or (3) simply did not want to engage in the hassles of participating in a contest.

Threadless also found that in the first year, over ninety percent of the buyers who made purchases through an Artist Shop website were shoppers who had never previously purchased a product on the Threadless website. In other words, they were new buyers attracted to different types of art and different artists who had never displayed on the crowdsourced website. Within a few years, the number of new buyers far outnumbered those with whom Threadless had already done business.

In lieu of the absence of major cannibalization, Threadless expected that it could continue its crowdsourcing platform alongside the Artist Shops without having much effect on Artist Shops. It also learned that many graphic artists continued to find value in interacting with the online community. The intrinsic rewards of winning continued to motivate some, as did the less tangible gains from winning. Many graphic artists liked the “forcing function” of a deadline for a contest, which motivated them to complete a piece. Many also liked employing themes built around specific topics.

At an operational level, Threadless experienced scope economies between the two platforms, and this is represented as overlapping operational processes in Figures 2B,C. Some of these processes Threadless had built up for itself, while some had been modified and packaged for licensees of Artist Shops. Overlapping processes included the payments functions, the tracking and monitoring of SKUs, the delivery tracking and monitoring, the communications with manufacturing partners, the quality control processes, and some of the marketing campaigns.

Not all went smoothly. For example, after difficulties at one manufacturer during the 2016 holiday season, Threadless learned the hard way about the value of secondary and tertiary sources of manufacturing. Management expanded its partnerships with additional manufacturers thereafter. That expansion in the capacity of potential supply accommodated what became a growing demand from buyers and an expansion in the scale of participation from graphic artists and others. In other words, over the next few years the number of manufacturers grew along with the broad growth in Artist Shops.

By 2020 Threadless had built up dozens of relationships for manufacturing items—shirts, pants, socks, and so on—and had integrated its own digital software into the machinery of these manufacturers and into their processes. The manufacturers received a design, an order, and an address for the destination of the item. They were responsible for timely printing, quality control, and shipping. Each relationship required customizing the interfaces to make the transmission of information seamless and free of friction between the partners. Once again, Threadless' own staff wrote the software after Threadless' management qualified a partner.

Developing a supply chain of this scale generated motives for an additional process innovation. It required routing software to translate the orders from an Artist Shop or the Threadless website into orders at a manufacturer. The software chose the best manufacturer or set of manufacturers for the order. This is a comparatively straightforward algorithm to develop when a buyer orders one SKU, since it requires the software to assess factors such as the geographic distances between the buyer and the manufacturer, the recent experiences with the quality of items from a potential manufacturer, the quantity of recent orders to this manufacturer in relation to their capacity, and whether they are a new supplier or one with a long history of reliable performance. It is also comparatively straightforward when the purchase is a large-volume order, unless the order exceeds the known capacity of a manufacturer. However, it is a more challenging algorithm to route a multi-item order that extends across multiple SKUs of different types of clothing for which no single manufacturer possesses all the required machinery. Again, Threadless built this algorithm to its own specifications, which is one of its most valuable processes.

Such a complex supply chain also required Threadless to monitor suppliers for quality, which was challenging due to the number of external partnerships. In addition to site visits, Threadless' employees make regular orders from the entire portfolio of manufacturers to assess quality of production.

More than 100,000 graphic artists license Artist Shops—albeit, <1% of the artists account for most of the sales^14^, and the sales from those far outweigh the sales from the crowdsourcing contests. While it was unsurprising that the sales experience among graphic artists displayed a skewed distribution, the skew between the sales experience of the Artists Shops and the crowdsourcing contests was less expected, and both imbalances created a challenge for supporting the small number of “superstars,” as well as the graphics artists who comprised the enormous “long tail.”

In response, Threadless had to co-invent again with the creation of a new job: They created a social media presence that directed traffic to specific websites, much like online advertising can direct traffic to Threadless' website. Established artists already had substantial profiles and merely needed to maintain them. Others had little or no social media following and needed to build it. Still others needed advice on how to respond to changing trends. In response, Threadless hired new employees, whose job entailed supporting and expanding the social media presence of artists.

In addition, Threadless hired a few employees to help handle the special challenges faced by its superstars, such as the crush of managing social media. Because these artists generated substantial sales, Threadless learned that helping popular artists also helped Threadless. Just as with other services, this is an opt-in service, and one that recognizes the effort it takes to coordinate modern social media accounts on Instagram, Twitter, Facebook, and elsewhere.

## Creating PODC in artist shops

What lessons emerge when we step back and view the entire narrative? What prompted pioneering in new business processes to support PODC? What enabled the pioneers in PODC to achieve a high volume of transactions and low cost PODC?

### Origins

Why did PODC emerge when it did? A simple answer stresses that high-quality machines became available only recently. That is too simple because machines alone were not responsible for the growth of PODC^15^. A deeper question asks why a successful electronic commerce platform emerged for PODC in 2016 at Threadless and not earlier.

The answer must stress that effective co-invention combines two distinct sets of information—insight into technical potential, and close familiarity with purchases of artistic clothing and workflows to support it. Threadless was familiar with these. In 2012, Threadless was well-placed to take advantage of the opportunity because its crowdsourcing business had already introduced the company to graphic artists and their buyers, which also motivated Threadless to develop some of the components required for PODC business. Importantly, Threadless also had already built up a level of trust with the online graphic artist community, as well as a level of operational skill with digital services.

Threadless' first actions were defensive, oriented toward incorporating digital printing into crowdsourcing and making the transition from the business process depicted in Figures 2A,B. This defensive motive led Threadless to PODC at an early moment in the transition of digital printing from low to high quality; and Threadless learned early on how to operate the process of licensing the winning design and printing it on clothing (e.g., shoes, pants, shirts, socks) and objects (e.g., cups, bowls, blankets and so on). Through such experience, it learned the basic elements of the PODC business. Because any design could be printed almost immediately after an order with no inventory needed, this reduced its warehousing costs. From there, the next co-invention steps led to a new platform, which again largely involved the creation of packaged services for graphic artists. These were riskier, but, as it turned out, these attracted a new group of graphic artists.

### Co-invention to support different motives

Threadless' defensive motivation for experimenting with PODC illustrates why invention on tangible technical equipment alone does not explain the success of PODC. Some of these co-inventions were comparatively straightforward for a technical team with experience in electronic commerce, such as designing a packaged format for a web page that enabled artists to choose among different features. Some were quite complex, such as the routing software to take a high volume of orders across a range of SKUs and distribute them among numerous partners. Integrating them into a bundle of services to be sold was no small feat, and drew on experience with order-fulfillment in the context of web-enabled electronic commerce. It required an understanding of both the capabilities of many digital processes and of PODC.

Such efforts drew on an understanding of the machinery capacity at partners, and it built on industry-specific knowledge about how to design software for business processes. As the motive changed, so too did the intangible assets on which Threadless drew. Another important intangible asset was the trust built up between graphic artists and Nickell, which generated a willingness to commit to a service from Threadless.

Packaging of services had to be co-invented. Many graphic artists did not possess the skills required to build an online business that printed their designs on any clothing. Many signed up for a service that not only provided that capability, but also gave access to PODC. Most willingly gave discretion to Threadless to do pricing for them because they Threadless gave them the option to withdraw. That led many graphic artists to trust Threadless more.

Yet not all artists wanted the same services, and Threadless learned to package those with useful boundaries. Key co-inventive acts in packaging were those that preserved the independence of the graphic artist and gave them control over their brand, while also laying out a menu of options for additional services, such as managing ad campaigns and sales. Other related aspects included the abundant support and advice Threadless gave to graphic artists, in which the company passed on lessons learned from experience^16^.

The benefits of the discretion also accrued unevenly across the platforms, as might be expected. An artist with a large existing following could accrue enormous benefits from the new potential to translate their art into merchandise. For such an artist, the emergence of Artist Shops in 2016 would lead to a financial windfall.

There is also some dependency and vulnerability built into Threadless' operating model. Supply-chain interruptions and global capacity limitations are a source of concern, and there is no co-invention yet that Threadless has created to reduce these risks to a negligible level. Shipping always imposes delays in gratification for the buyer, so Threadless must manage these risks.

### Pricing and co-invention

As was described previously, one of the options Threadless offered its artists was to price the products for them in Managed Shops. To appreciate co-invention in this dimension, begin the analysis from a position of skepticism. It seems utterly plausible that the graphic artist knows more about demand for their own designs, and which to price high and which to discount. How is it possible that Threadless' pricing is superior to letting graphic artists retain discretion?

Consider what Threadless cannot know. Threadless cannot know how much the artists value their own products, how much effort went into creating them, which designs tend to receive more interest from close friends, which designs touch on themes that an artist's fanbase would most appreciate, nor how urgently an artist does or does not want to sell specific items. With tens of thousands of clients, Threadless' management is not in any position to be informed about most aspects that graphic artists would know about their own art, nor about the preferences of the artists' fan bases. In short, Threadless is uninformed about all the nuances of demand for the artist's work. At best, it can implement an algorithmic rule that prices pieces without accounting for each supplier's idiosyncratic preferences and situation. How could it be the case that such decision making over pricing appealed to so many graphic artists?

Every answer highlights the same type of trade-off. Although Threadless was likely to err by *not* accounting for much of what the artist knows and wants, these errors were small in comparison to the benefits artists received when they gave Threadless the discretion. Overall, therefore, artists perceived that Threadless' pricing algorithm improves upon any alternative in which the artist retains discretion over selling.

What benefit does Threadless bring to the decision? Consider open questions about pricing over days and time of day. What results in higher revenue on the weekend, higher or lower prices? Would a flash sale from 6 pm to midnight yield extra revenue? If so, on which days are such sales most effective? These are questions that Threadless can answer with its considerable experience. It has information about the sales patterns during different days and different times of the day on its own site. It also has experience with engineering flash sales on different days and times to take advantage of different patterns of traffic from sampling its own web site.

In addition, Threadless has specialized knowledge from selling a broad range of items for all the different graphic artists. Graphic artists have only limited time spend learning how demand fluctuates around holidays, days of the week, and times of day. Although each graphic artist may have an idiosyncratic experience, most of the graphic artists have little or no idea how to modify their prices accordingly, while the management at Threadless has both considerable experience and information about what has worked. Indeed, most graphic artists know little about the factors that shape pricing and might not price appropriately. Such errors could cost the graphic artist considerable sales, as well as produce losses.

Another subtle feature of Threadless' service comes from simplified pricing—that is, the reduction of price dispersion across similar items. Simplified pricing benefits the artist by reducing buyer's confusion from menus, which is a real possibility after the explosion of SKUs. More subtly, Threadless' pricing service results in near *uniform pricing* on its website, which reduces price comparisons and simplifies a user's ability to compare across options of combinations of art and clothing items. Uniform pricing also fosters overall commitment. Its continued use implicitly promises to repeat users that the uniform pricing will continue. This makes an Artist Shop, as well as Threadless' website, more inviting for a revisit, and *all* graphic artists benefit from encouraging more potential buyers.

Threadless' pricing service also helps artists avoid a common error made by artists with little experience in sales. As it turns out, bringing designs to market can be a hassle for many artists, who might then price their services to reflect their own efforts. The value of the design is determined by its appeal, not by how difficult it was to put together, nor how long it took, nor any other aspect of the artist's inconvenience. Threadless' program avoids the error of using the artist's effort to benchmark how to price an item with that design.

As earlier noted, Artist Shops require that artists trust Threadless to look after their interest. That remains doubly so with the pricing feature of Managed Shops. This is one way in which the intangible asset, trust, plays an essential role in the service.

### Free co-invention is not free

It costs nothing to replicate a design on pieces of additional clothing of different sizes, and it costs nothing to replicate it on a variety of objects. That translates into no cost to satisfy the personal preferences of a buyer for a combination of object and art. That also translates into no cost to make a portfolio of items with identical art.

Greenstein and Nagle (2014) propose a label, *digital dark matter*, for some innovative building blocks of the digital economy with zero cost. Digital dark matter are digital goods and services that have no monetary value, are effectively limitless, and serve as inputs into production.

For Threadless, a combination of technical and business innovations in a single platform made the creation of digital dark matter possible. Through its co-invention and investments, Threadless had realized economies of scale that made it possible to purchase and maintain a PODC machine. This was because Threadless had aggregated enough user demand of enough artists with enough buyers for enough products that it could use a PODC machine and change from one print to another with merely incurring the incremental costs of ink and time. The low cost also arose from an ordering process that incrementally cost nothing to use. The software could operate for each order at no effective cost. Together, these resulted in a production and ordering process where the incremental costs of another order were effectively zero and were limitless. In other words, they are digital dark matter.

There is a sense in which PODC is not free, however. To realize the economies of scale from high volume matching, Threadless was required to ship the product to the user. Shipping costs are always non-zero. In this sense, buyers always must pay a fixed fee to access an unpriced attribute of the service. Similarly, Threadless cannot offer this service for no costs. It must cover the operational costs that make it available to buyers.

Zero-cost reproduction of art on a range of clothing is not an innovation whose benefits will register in GDP. The value of a t-shirt in GDP is the value of the revenue. That is so whether it is plain white or dyed, generic or personalized. Its sale represents its contribution to GDP. The introduction of high-quality PODC will not lead to massive measured gains in productivity or GDP, because a more beautiful piece of artful clothing just raises sales at Threadless and decreases sales at another firm. It is an innovation that enables “business stealing” from other clothing firms and does not expand the potential for new production levels.

To say it succinctly, neither improvement in input nor the improvement in user satisfaction are measured by tangible action. That does not mean the innovation lacked value, and it does not imply that buyers are not happier with their purchase than any alternative. It merely implies that standard measurement is inadequate for measuring the gains to personalized clothing.

### Network effects and benefit of co-inventing early

Once it achieved scale, did Threadless' platform contains features that make it self-perpetuating due to network effects? Yes, and in this case, the platform displays two distinct types of network effects. It contains cross-side network effects and same side effect.

Cross side network effects are those in which more participation by one side—say, graphic artists—induces more participation by another—say, buyers—and that supports more activity by another—say manufacturers of a product. Manufacturer participation is particularly important, because its growth supports two dimensions of the platform, volume of production and breadth of products. The volume and breadth generate advantages for Threadless.

At the time of this writing, the Threadless platform has exceeded any minimal size required to induce manufacturing of any product desired by Threadless' management, so the network effect enhances the breadth of their product line. In addition, the joint participation of so many participants create a high likelihood of it all persisting. All sides have invested in making their activities work seamlessly with Threadless' platform, and the persistence of one motivates the other to stay. Now that it is large, these network effects make the whole less likely to diminish in size.

The platform also displays some same side network effects, and these are mostly affiliated with its scale, though these appear to be somewhat limited. For example, selling many products of many graphic artist on Threadless' web site makes it focal for some buyers, and that induces more participation from additional graphic artists, who bid for business from some of the buyers attracted to the site. The scale of participation increases the competition too—in the sense that it gives buyers more options. For graphic artists with an established brand (e.g., Strange Planet), this competition would be negligible, and the additional distribution channel would give them additional contact with users they may not otherwise reach. For graphic artists without an establish brand, the additional contact with users is valuable as well, and in spite of the competition. These gains are limited, however, by the ability of graphic artists to distribute their product themselves.

Now that a workable solution has been demonstrated, it practically invites competitors. An open question is whether platforms in electronic commerce could do sometime similar and take a bite out of the market share for PODC. At slightly lower levels of scale—e.g., sellers on Etsy –already offer some PODC services, so some level of competition is inevitable. In addition, it seems pretty obvious that a big content owner with many trademarks and copyrights and sufficient volumes, such as Disney, could organize PODC themselves and cut out the intermediary. But what about other players? While the technical dimensions are not beyond many established firms, how would they do organize this activity if graphic designers do not trust them? As of this writing, this is an open question.

## Conclusion: An archetype of innovation within platforms

A reductionist view of the long history of innovation in textiles production during the last two centuries might observe that invention aims to achieve more scale, less cost, and better color, but quite often improving the first two comes at the expense of color and artistry. The power loom, for example, initiated the automatic weaving of colored cloth near the end of the eighteenth century. Soon after, the Jacquard machine emerged, which made it possible to weave many geometric designs with no negative change in scale. There designs themselves, however, were limited to patterns. Seen against this broad history, the emergence of PODC today continues a long quest to maintain the low costs of production at scales without sacrificing the option to customize and beautify. In brief, PODC enables an abundance of color and design across a breadth of clothing and items, and at a low expense never before achieved.

What co-invention led to PODC? Closely examining the actions of a leading firm shows that an external event generated focused search for incremental changes to existing operations. PODC emerged from two distinct sets of actions, which results in two related clusters of co-inventions. The first set was defensive and informative, oriented around improving the processes at a crowdsourcing site by adopting digital printing, and saving expenses in anticipation of a future event, which the management interpreted as a threat. As it was learning from these first experiences, the firm partially altered its relationship with a key business partner, graphic artists, including changes to the governance of intellectual property.

Then management considered a second set of co-inventions. The second set was imaginative and entrepreneurial, oriented around developing a new platform to address an unmet need of both graphic artists and potential buyers. This included the redesign of the boundary of discretion between the platform leader and the graphic artists, which, in turn, redefined the relationship between buyer and seller. Key features of the transaction between seller and buyers, such as the pricing, became coordinated by the platform.

The old and new platforms made use of overlapping processes for digital printing, and, as it turned out, created little cannibalization among sources of revenue. The lack of cannibalization occurred because the new platform created a market transaction that appealed to more than just the original participants on the old platform. It attracted participation from new graphic artists and their buyers. Though Threadless did not anticipate it, after the fact, we see the two platforms—one oriented toward crowd-sourcing graphic art, and one oriented toward PODC—were largely not in conflict with one another, enabling Threadless to escape a disruptive firestorm.

This analysis provides insight into the management's perspective prior to the emergence of a new platform. If similar trends emerge from additional studies of platform innovation, it should not come as a surprise that much of the value of leading platforms in the modern economy arises from business process co-inventions.

An important open question is whether two suppliers always perceive the setting in the same economic terms, and aggregate up to a similar level of economic incentive to invent a new platform. Another open question is whether a similar hierarchy of business process inventions would emerge in another setting—starting with those less risky and complementary to the core invention and ending with those more distant but related to the needs of the other platform partners. This study also suggests there would be insight from comparing co-invention across suppliers, and analyzing whether leading supplier are earliest to co-invent when new invention emerges. Those open topics await further studies.

What features generalize beyond this example? Could a similar platform emerge in other areas of art where digitization has overtaken the medium? Text and photography both seem ripe for a similar operating model. So too does any activity that uses 3D modeling and printing, where, again, the potential for recombination and value creation are high among many applications in product manufacturing and prototyping, architectural modeling and demonstrations, and artistic sculpture, to name a few. Though thoroughly digital at this point, it is difficult to see a similar platform emerging in music or movies, both because these retain complex copyright regimes and because the potential for recombination would be rather different than the operating model described in this study. These are, of course, open questions, unless or until entrepreneurs answer them by either building viable businesses or by doing the opposite, namely, trying to do build a business and failing to find a profitable operating model.

## Data availability statement

The original contributions presented in the study are included in the article/supplementary material, further inquiries can be directed to the corresponding author/s.

## Author contributions

The author confirms being the sole contributor of this work and has approved it for publication.

## Conflict of interest

The author declares that the research was conducted in the absence of any commercial or financial relationships that could be construed as a potential conflict of interest.

## Publisher's note

All claims expressed in this article are solely those of the authors and do not necessarily represent those of their affiliated organizations, or those of the publisher, the editors and the reviewers. Any product that may be evaluated in this article, or claim that may be made by its manufacturer, is not guaranteed or endorsed by the publisher.

## Footnotes

^1^We were fortunate to collect interviews from Threadless' executives, who graciously agreed to answer questions for two Harvard Business School case studies (Lakhani and Kanji, 2008; Greenstein et al., 2021). This essay builds on these and refers the reader to it for many details.

^2^Throughout the narrative *intangible assets* are assets that do not have a physical component. The narrative uses the term broadly to include copyright licenses, the software that supports daily operations, and the level of trust between Threadless and graphic artists.

^3^It is worthwhile to appreciate these tensions in some detail as they will illuminate which intangible assets were most valuable and why changes to the discretion ceded to graphic artists was so innovative.

^4^Take an early year in the era of screen printing. In 2007, for example, the material cost of t-shirts amounted to $7 per shirt. The corresponding average price per shirt amounted to $23m/1.5m = $15.3 per shirt. Rewards for contests add approximately $0.60 per shirt. See Lakhani and Kanji (2008), pages 2 and 3.

^5^When Threadless first began its reward was $100 for winning a contest, then $250, and eventually $2,000.

^6^Threadless followed the sentiments of the online crowd most of the time, but occasional irregularities with voting or other unexpected complications with sampling the opinion of the crowd could interfere with the recommendations of the vote.

^7^See Lakhani and Kanji (2008).

^8^While at first Threadless did perform all the manufacturing itself, it did not persist with this choice. It eventually developed relationships with partners for screen-printing.

^9^Somewhat remarkably, the team is comparatively small, comprised of ten or fewer employees. The narrative illustrates just how much a programming team with competence at the frontier could accomplish over a medium time horizon with visionary leadership.

^10^For the sake of brevity this narrative does not recount them, but see Greenstein et al. (2021) for more information.

^11^https://artistshopshelp.threadless.com/article/689-what-are-base-cost, accessed September 2021.

^12^This selection process was carried out at the shop level, so an entire shop's collection was either included in the marketplace or not. For these items Threadless compensated from a different baseline cost, and it was typically incrementally higher than the cost for sales on an artist shop.

^13^The amount paid to artists for these deals varies, but at a minimum it comes to 20% of earnings.

^14^In 2020, the top ten shops accounted for one-third of Artist Shops' overall revenue and the top 100 accounted for 80% of the overall revenue.

^15^Farag (2021), presents an amusing perspective on some of the recent choices available to a small entrepreneur. This situation changes frequently, so up to date information can be difficult to acquire. Wikipedia is as good as any other source for a sense of the array of options. For example, see https://en.wikipedia.org/wiki/Digital_textile_printing, or https://en.wikipedia.org/wiki/Direct-to-garment_printing, or https://en.wikipedia.org/wiki/Dye-sublimation_printing, (accessed September 2021). Among the many digital printers for large scale production, the following printers are referenced, including, for example, Atexco ATPColor.it, Roland, Durst, Hollanders Printing Systems, Vutek, KERAjet, Reggiani, MS, Osiris, Stork (later SPGPrints), Konica-Minolta, and Zimmer. The listing of so many firms is symptomatic of the many available options and trade-offs facing a manufacturer when selecting among equipment.

^16^ https://artistshopshelp.threadless.com/category/678-launch-guide
